# An mHealth App (NeoMayor) to Promote Healthy Lifestyles and Brain Health in Older Adults: Design and Validation Study

**DOI:** 10.2196/71936

**Published:** 2025-10-01

**Authors:** Myriam Gutiérrez, Victoria Cabello, Carol D SanMartín, Jorge Mauro, Gada Musa, Melissa Torres, Maria Consuelo San Martin, Carlos Márquez, Rodrigo Beltrán, Nicole Rogers, Felipe Salech, Daniela Ponce, Christian Ampuero, Pia Varas, Bastián Gamboa-Labbé, Víctor David Cortés, Carlos Vega, Rocío Ruiz, Juan Velásquez, Rodrigo Vergara, Maria Isabel Behrens, Jamileth More, Carolina Delgado Derio

**Affiliations:** 1 Unidad de Cerebro Saludable Hospital Clínico Universidad de Chile Santiago Chile; 2 Facultad de Medicina, Departamento de Neurociencia Universidad de Chile Santiago Chile; 3 Núcleo Magíster en Salud de la Mujer, Facultad de Medicina y Ciencias de la Salud Universidad Mayor Santiago Chile; 4 Facultad de Salud y Odontología, Escuela de Kinesiología Universidad Diego Portales Santiago Chile; 5 Escuela de Psicología, Universidad de Humanismo Cristiano Universidad de Humanismo Cristiano Santiago Chile; 6 Centro de Investigación Clínica Avanzada (CICA) Hospital Clínico Universidad de Chile Santiago Chile; 7 Instituto de Nutrición y Tecnología de los Alimentos (INTA) Universidad de Chile Santiago Chile; 8 Escuela de Psicología Universidad de Los Andes Santiago Chile; 9 Departamento Medicina Interna, Facultad de Medicina Universidad de La Frontera Temuco Chile; 10 Instituto de Neurocirugía Dr. Alfonso Asenjo Santiago Chile; 11 Clínica Universidad de Los Andes Santiago Chile; 12 Web Intelligence Centre (WIC), Departamento de Ingeniería Industrial Universidad de Chile Santiago Chile; 13 Complex Engineering System Institute Santiago Chile; 14 Departamento de Kinesiología, Facultad de Artes y Educación Física Universidad Metropolitana de Ciencias de la Educación Santiago Chile; 15 Centro Nacional de Inteligencia Artificial (CENIA) Santiago Chile

**Keywords:** mobile health, mHealth, healthy aging, noncommunicable diseases, brain health, dementia prevention

## Abstract

**Background:**

In Latin American countries, the prevalence of noncommunicable diseases has increased rapidly in recent decades. Mobile health (mHealth) apps are now widely available at low cost and are easy to implement, offering an opportunity to encourage healthy lifestyles in older adults. However, at present, there are no mHealth apps that integrate multidomain healthy lifestyle interventions specifically adapted for older adults in Chile.

**Objective:**

This study aims to describe the development and validation of NeoMayor, an mHealth app designed to promote healthy lifestyles as well as cardiovascular and brain health in older adults in Chile.

**Methods:**

NeoMayor was developed iteratively with feedback from users and input from a multidisciplinary team of clinicians, researchers, and software developers. Using lean user experience methodology, we ensured user involvement throughout the design and validation process. The research was conducted in 2 phases. In the design and development phase, we created and adapted evidence-based recommendations. In the validation phase, we conducted a pilot study to assess usability, adherence, and cardiovascular health (CVH). A total of 60 functionally independent and cognitively healthy participants used the NeoMayor app for 2 months. Clinical and cognitive assessments were conducted before and after app use. We held 26 cocreation sessions with users, consulted experts, performed a literature review, and collaborated with a team of app developers to create a functional prototype.

**Results:**

The mean age of the participants was 71 (SD 15) years, and 85% (51/60) were female. Participants had an average of 9.8 (SD 3.6) years of education. At the end of the 2-month intervention, usability testing indicated high engagement and adherence, with participants using the app for an average of 6.6 (SD 11.85) minutes per day twice a week. Improvements were observed in global CVH, with the mean Life’s Essential 8 CVH index score increasing from 64 (SD 10) to 68 (SD 11; *P*<.001). Reductions were noted in systolic blood pressure (10 mm Hg) and waist circumference (7 cm), along with increased high-density lipoprotein cholesterol levels. Participants also showed improvements in self-reported physical activity and diet, higher scores on the Short Physical Performance Battery, and faster performance times on the sit-to-stand and gait speed tests. The app was optimized for broad compatibility with Android devices, safe data collection and storage, and compliance with data privacy regulations following good clinical practices. The final product is ready for testing in a randomized controlled trial.

**Conclusions:**

This study represents the first initiative in Chile to develop and validate an mHealth app to promote healthy lifestyles as well as cardiovascular and brain health in older adults, offering an effective, accessible, and affordable solution for promoting healthy aging in Latin American countries.

## Introduction

### Background

Accelerated population aging in recent decades, particularly in low- and middle-income countries, has led to an increase in the prevalence of noncommunicable diseases (NCDs) in adults [1,2]. Among people aged ≥70 years, cardiovascular diseases and dementia are now the leading causes of disability [3,4]. In Latin America and the Caribbean, dementia prevalence is projected to rise by 200% to 300% over the next 30 years [5]. Globally, an estimated 80% of cardiovascular disease risk factors are potentially modifiable [5,6]; in Latin America and the Caribbean, approximately 54% of dementia risk factors are also potentially modifiable [7]. This highlights the urgent need for preventive initiatives aimed at reducing the burden of cardiovascular diseases and dementia [8].

Increasing evidence supports the effectiveness of multidomain healthy lifestyle interventions—including physical activity, healthy diet, cognitive stimulation, social engagement, and health control—for treating and preventing NCDs, reducing stroke risk, and enhancing cognitive reserve [9-11]. In Chile, despite the existence of public primary prevention programs for NCDs, there has been a significant increase in the prevalence of diabetes and obesity in the past decade [12,13]. These unfavorable outcomes of Chilean prevention campaigns could be attributed to their limited outreach; low population adherence; and multiple implementation challenges [14], including health care inequities [15].

To address this implementation gap in health promotion, mobile health (mHealth) platforms have emerged as promising tools. mHealth encompasses medical practices supported by mobile devices such as smartphones, tablets, and wearable sensors. These technologies are easily accessible, widely available, have affordable costs, and are easily implementable [16-18]. Recent evidence supports the effectiveness of web-based and smartphone interventions in promoting cardiovascular health (CVH) in adults [16,19], providing insights for the design of user-friendly and accessible health technologies for older adults [20,21]. In high-income countries, numerous initiatives have been developed to promote healthy lifestyles through the use of mHealth [22]. Although many of these mHealth solutions can be easily adopted by young adults, older adults face several barriers to using mHealth, reducing its effectiveness in this demographic group [21,23]. These barriers include lower digital literacy and technological skills [24], physical and cognitive limitations [21,25], lack of trust and privacy concerns [26], and cost and technology access [23]. Regarding technology access, in Chile in 2022, 65% of older adults owned a smartphone (rising to 82% among people aged 60-69 y), and 60% had home internet access (rising to 90% among those living with younger people) [27-30]. Educational level is the most important factor related to technology use in Chile’s older adults, possibly associated with technology use skills and technology access [29]. Therefore, the design of mHealth tools targeting older adults should incorporate a user-centered approach that accounts for their physical and cognitive capacities as well as their technological skills [31]. Prior research recommends solutions that are intuitive, easy to navigate, supported by health care professionals, and accessible, for example, apps with large interfaces and simplified interactions [32,33]. However, in Chile, despite relatively good smartphone access, there are currently no mHealth solutions for promoting healthy lifestyles that are adapted to older adults. Our main hypothesis was that health care content delivered through a mobile app—cocreated using participatory design methodologies and based on a multidomain intervention approach—would be easy to use and would improve CVH in older adults.

### Objectives

We aim to describe the development and validation of NeoMayor, a mobile app cocreated with older adults to promote healthy lifestyle habits, support NCD management, and enhance cardiovascular and brain health in this population.

## Methods

### Study Design

**Figure 1:**
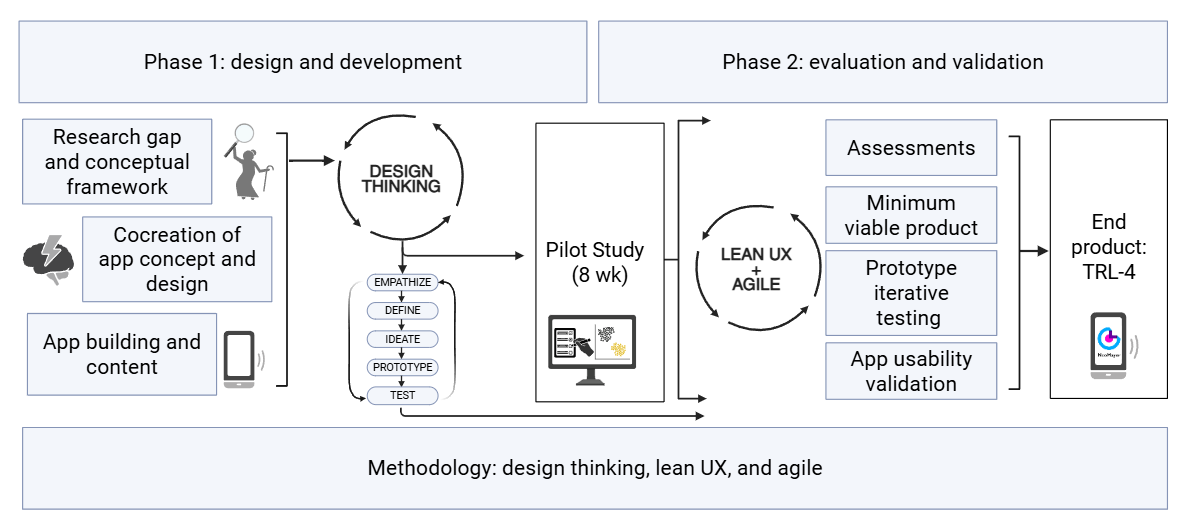
The 2 phases of the NeoMayor app study. RCT: randomized controlled trial; TRL: technology readiness level; UX: user experience. Created with BioRender [34].

The development and validation of NeoMayor were planned using a user-centered design approach [31]. The study was conducted in 2 phases: phase 1 (design and development with older adults, guided by lean user experience [UX] methodology) and phase 2 (prototype validation through a 2-mo pilot study in which participants used NeoMayor on their smartphones). The entire process lasted 26 months, from February 2022 to April 2024 (Figure 1 [34]).

### Phase 1: Design and Development

The NeoMayor app was developed to promote healthy lifestyles as well as cardiovascular and brain health in older adults in Chile. From the outset, the app was designed to provide personalized lifestyle recommendations based on each user’s baseline health status. Its architecture includes a secure electronic database compliant with clinical data privacy standards [35].

**Figure 2:**
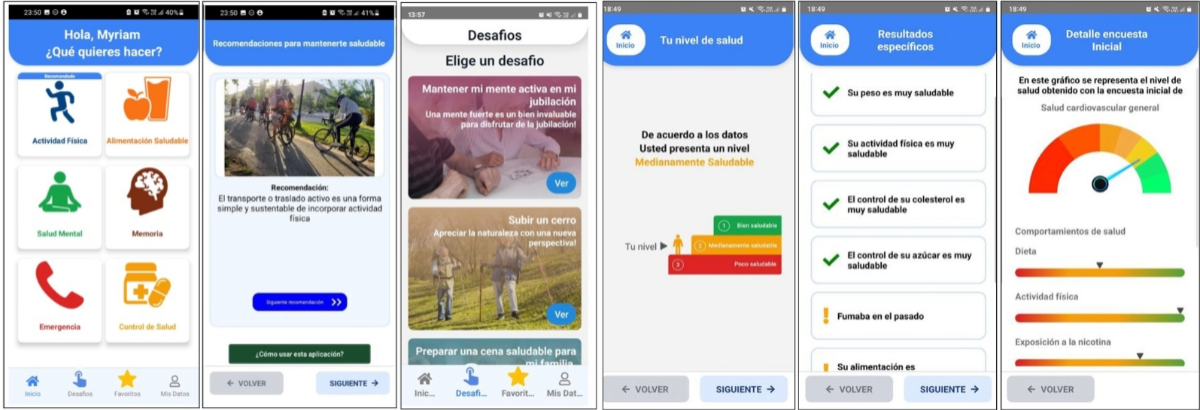
Screens of the NeoMayor app. (A) Categorization of cardiovascular health. (B) Initial menu and goal-directed navigation.

The clinical multidisciplinary team, which included experts in neurology, geriatrics, physical therapy, nutrition, psychology, and biomedical sciences, led the creation of the NeoMayor app and collaborated closely with software developers to design and program the software. Using a design thinking process, we first identified user needs (empathy approach) and analyzed existing health technologies (definition approach). After gathering theoretical information, hypotheses were formulated, and proposed content from the clinical teams was validated by expert peers. Content creation was based on multiple guidelines, such as the World Health Organization (WHO) Integrated Care for Older People (ICOPE) handbook [36], the WHO guidelines for risk reduction of cognitive decline and dementia [36,37], the Chilean Cardiovascular Health Program [38], and the American Heart Association’s Life’s Essential 8 [39]. Life’s Essential 8 provides self-care recommendations to manage 8 modifiable risk factors for cardiovascular disease by adopting healthy lifestyle behaviors: eat better, be more active, quit tobacco, get healthy sleep, manage weight, control cholesterol, manage blood glucose, and manage blood pressure. Each risk factor can be measured on a semiquantitative scale from 0 (poorest) to 100 (healthiest), with the average corresponding to the global CVH index [25]. We selected the CVH index as our primary outcome because it has been validated as a predictor of cardiovascular events [40], brain health, and dementia risk [41]. In addition, because it comprises only modifiable risk factors, the CVH index can change over time, making it suitable for tracking progress in CVH [39]. We adapted the CVH index into a mobile questionnaire that provides feedback on health status and tailors recommendations accordingly. Participants are categorized as optimal (score: 79-100), moderate (score: 50-79), or poor (score: <50), using graphic images (Figure 2A). The app recommends periodic assessments to monitor changes in CVH over time.

The lifestyle content is structured into 6 modules: physical activity, healthy eating, mental health [42,43], memory, health control [6,38,39] (Figure 2B; based on guidelines from the WHO [44,45], the Chilean Ministry of Health [38], and Life’s Essential 8 [39]), and “emergency” (incorporated at the request of participants, who wanted quick access to emergency numbers and official resources on legal and health benefits). Content is delivered through video, audio, and educational images. Currently, the app features a repository of 33 videos, each approximately 5 minutes long; 30 audio recordings, each lasting 15 minutes; and 50 educational images. The app includes 2 navigation options: the first is free navigation, in which users can choose from 6 modules on the menu screen; and the second is challenge-based navigation, in which users set a specific goal (eg, “being able to climb a hill”), prompting customized, targeted training suggestions that combine content from multiple modules over a defined period (Figure 2B).

The next step was NeoMayor’s iterative refinement and development, guided by a combination of lean UX [46], agile [47], and design thinking methodologies [48]. The selection of lean UX and design thinking was intentional, given their strong alignment with the specific challenges and needs of older adults. These user-centered frameworks facilitate continuous feedback integration, empathy-based exploration of UX, and rapid prototyping, all of which are essential for optimizing usability and ensuring the app’s relevance for older adults with medium to high digital literacy [49]. Lean UX enabled rapid iteration to refine accessibility features such as color contrast, touch target size, and interface clarity [50]. In parallel, design thinking fostered cocreation through structured empathy and ideation stages, ensuring that the final product resonated with the lived experiences of older users [51]. Low-fidelity prototypes of the NeoMayor app were iteratively refined to emphasize accessibility, intuitive navigation, and content clarity.

The development team followed agile Scrum practices, with biweekly sprints and sprint retrospectives involving clinicians and software engineers. Tasks were managed using Trello, and code repositories were version-controlled using Git and GitHub. Continuous integration was achieved using GitHub Actions, which facilitated automated testing and deployment to staging environments before release to the Play Store.

As part of this cocreative and coconstructive process [52], we implemented several interface adjustments, including high-contrast colors, large touch targets, simplified gestures, and accommodations for motor and cognitive needs. This combined approach enabled the development of a tool that is not only clinically robust but also practical, intuitive, and acceptable to older adults across a spectrum of technological familiarity. Classification algorithms and user surveys further guided customization of the UX.

**Figure 3:**
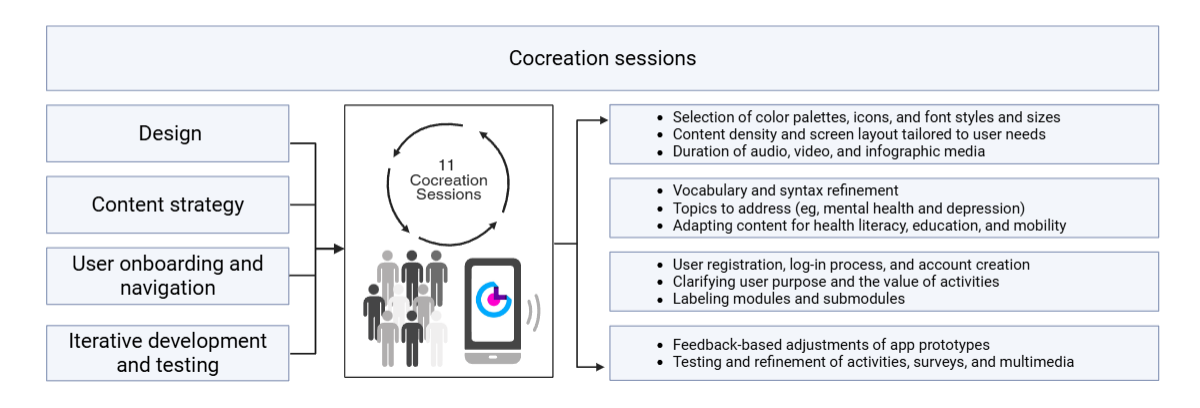
The NeoMayor iterative participative design process. Created with BioRender [53].

We held 26 cocreation sessions with 30 older adults (female: n=21, 70%; male: n=9, 30%; age: range 60-75 y) to validate the language, content, and user interface elements (Figure 3 [53]). These sessions provided critical feedback on layout, navigation, and instructional clarity, which shaped the app’s design.

After the content was finalized, the software development team generated both front-end and back-end components. The app was built using the open-source React Native framework for cross-platform compatibility on Android devices and a Django backend on servers maintained by the Web Intelligence Center for secure data handling. Once compiled with Gradle, the NeoMayor app was validated through the Google Play Console and released on the Google Play Store as a downloadable app.

The app deployment was managed through the Google Play Console, with version tracking and staged rollouts to monitor performance and crash reports. Updates were issued using semantic versioning (eg, v1.0.0) and were preceded by internal QA cycles. A dedicated beta testing group of older adults provided feedback on each release candidate.

Throughout the iterative design process, the team (users, clinicians, designers, and engineers) validated the app’s content, navigation flow, and overall UX. As suggested by pivotal software development literature [54], we conducted micro-usability tests to evaluate ease of navigation, icon comprehension, and text readability. These tests provided insights on interface improvements, leading to high-contrast designs, labeled icons, and enlarged touch targets (Figure 3). Thus, the NeoMayor app was evaluated at the prototype level in terms of usability, functionality, and efficacy, achieving positive results across all evaluated domains. After 12 months, we obtained a minimum viable product: a mobile app that classifies users according to CVH and mobility and offers personalized content organized into 6 modules.

### Phase 2: Evaluation and Validation (Pilot Study)

#### Participants

**Figure 4:**
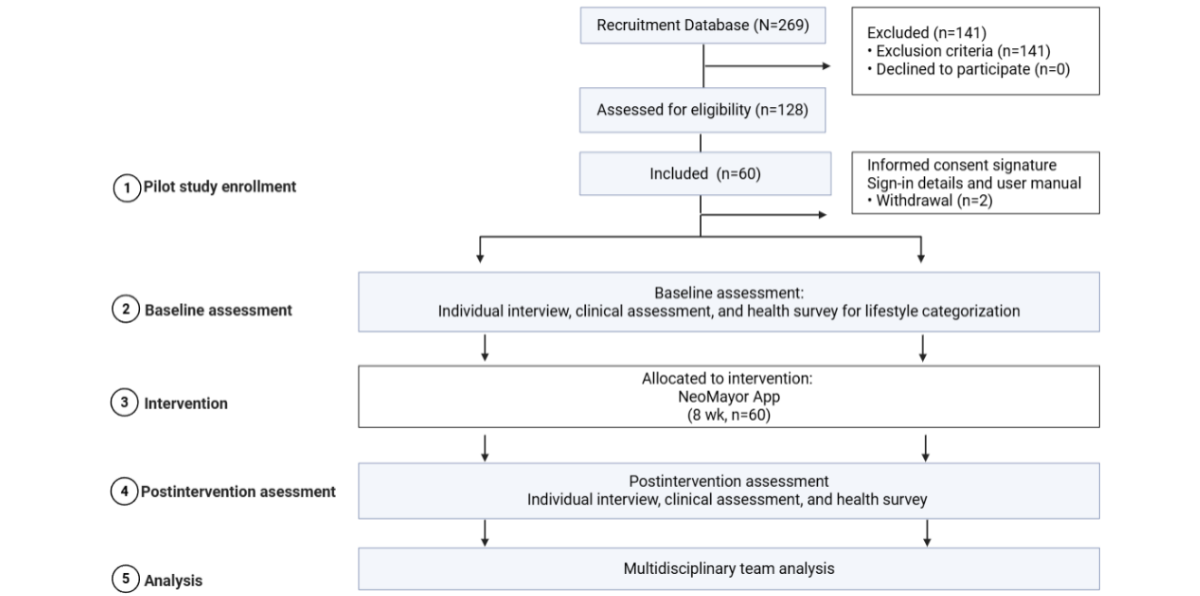
The NeoMayor pilot study flowchart. Created with BioRender [55].

We recruited functionally independent older adults aged 60 to 77 years living in Santiago, Chile, who were able to read, write, and use technology (digital skills sufficient to independently use a mobile smartphone; Figure 4 [55]). Participants were recruited through voluntary sign-up notices from ambulatory programs at the Hospital Clínico de la Universidad de Chile, civil society representatives of the Servicio Nacional de Personas Mayores, and the advisory committee of the Instituto Nacional de Geriatría. Printed invitation posters were also distributed in various community and public spaces across Santiago. To qualify for inclusion, participants were required to have a moderate to high cardiovascular risk, defined as the presence of at least three of the following cardiovascular risk factors: (1) hypertension (blood pressure >140/90 mm HG or receiving pharmacological treatment), (2) diabetes or fasting glycemia level of >100 mg/dL, (3) obesity (BMI ≥30 kg/m^2^), (4) current smoking status, and (5) elevated total cholesterol level (>240 mg/dL). In addition, participants had to be free of dementia, defined as a total score of ≥8 on the memory, fluency, and orientation test, which is a 5-minute screening test for cognitive decline validated for the Chilean population [56]. The exclusion criteria included significant impairments in independent mobility, substantial auditory or visual impairments, neurodegenerative diseases, untreated psychiatric disorders or psychological diagnoses, and the presence of a severe or terminal illness. Participant recruitment began 1 month before the initiation of the pilot study. Different participants were involved in the 2 phases of the NeoMayor design: those engaged in cocreation sessions, design, and development (n=30), described in the previous section; and those who participated in the pilot study (n=60).

#### Pilot Methodology

A database of 269 individuals who expressed interest in participating in the pilot study was established, and we contacted 128 (47.6%) by telephone to assess their eligibility based on the inclusion and exclusion criteria. Of these 128 individuals, 62 (48.4%) met the inclusion criteria. Subsequently, 2 (3%) of these 62 participants withdrew voluntarily for personal reasons, leaving a final sample of 60 (97%) participants. Participants then underwent face-to-face interviews and received a complete clinical, physical, and cognitive assessment at baseline and after the 2-month NeoMayor app use period. Trained professionals (physicians, physical therapists, and psychologists) performed the assessments. For consistency, the same evaluator performed the pre- and postpilot assessments, blinded to adherence results. The clinical assessment lasted approximately 1 hour and included a structured interview to collect demographic and sociocultural data, current health conditions, and medication use; measurement of biometric data, including anthropometry; assessment of blood pressure; blood tests (glucose and lipid levels); and structured questionnaires to assess lifestyle habits adapted for Latin America [11]: the Pittsburgh Sleep Quality Index [57], the Mediterranean-DASH Diet Intervention for Neurodegenerative Delay Diet Scale [58], and the International Physical Activity Questionnaire [59]. By using these data, we calculated the CVH index, including the total score and 8 subscores [39]. The cognitive evaluation included the administration of the Mini-Mental State Examination [60]; the Geriatric Depression Scale [61]; self-perception of memory scales (MCA) [62]; and the memory, fluency, and orientation test, which is a 5-minute screening test for cognitive impairment [56]. The physical performance assessment included the use of the handgrip test; the 5-repetition chair stand test, as recommended by the WHO ICOPE handbook [36]; the timed up and go test (both simple and dual tasks); and the Short Physical Performance Battery (SPPB) [63].

The NeoMayor app was installed on participants’ smartphones, and they received both training in its use and a printed user manual. For those who did not have a smartphone, the project provided one. All participants used the NeoMayor app for 2 months. In case of significant problems with the app or smartphones, assistance was provided. After 3 weeks, we contacted half of the users (30/60, 50%) via telephone to address any problems in using the app. Finally, to evaluate the pilot implementation, we used the RE-AIM (Reach, Effectiveness, Adoption, Implementation, and Maintenance) [64] methodology. At the conclusion of the pilot study, all participants completed surveys to provide qualitative data about usability, user satisfaction, and content preferences. Further assessments will be included in a randomized controlled trial (RCT).

#### Data Management

The data were collected in a REDCap (Research Electronic Data Capture; Vanderbilt University) database. All data analyses were conducted using R (version 4.0.0, 64-bit; R Foundation for Statistical Computing) and SPSS software (version 27.0; IBM Corp). After checking the distribution of the data, we performed descriptive statistics. Continuous variables were expressed as mean (SD) and 95% CI, and categorical variables were reported as percentages and 95% CIs. The potential effect of NeoMayor app use on participants’ CVH was measured using a paired sample *t* test comparing the CVH index before and after app use (within-group analysis) [65]. We calculated the effect size of within-group changes by using Cohen *d.*

Finally, we categorized the participants according to whether they showed improvement. Improvement was defined as significant if the difference between the CVH index before and after app use was >1 SD from the baseline CVH index. We then performed a binary logistic regression to establish which variables were associated with the improvement, using improvement (yes or no) as the dependent variable, with age, educational level, sex, baseline depressive symptoms, and baseline CVH index as independent variables.

### Ethical Considerations

The study was approved by the scientific and research ethics committee of the Hospital Clínico de la Universidad de Chile (Acta 36, OAIC 1220/21, FONDEF ID21I10096). All study participants provided signed informed consent.

## Results

### Demographic and Biomedical Data

A total of 60 older adults were included in the pilot study. The participants’ mean age was 71 (SD 15) years, and they had an average of 9.8 (SD 3.6) years of education. Of the 60 participants, 51 (85%) were female. Most of the participants used the internet at home (57/60, 95%) and owned an Android smartphone (49/60, 82%). We provided a smartphone to 11 (18%) of the 60 participants (n=7, 64% owned an iPhone; n=4, 36% did not have their own smartphone, or their smartphone was too old). The baseline characteristics of participants are detailed in Table 1.

**Table 1:** Participants’ baseline characteristics (n=60).

Characteristics			Values
Age (y), mean (SD)			71 (15)
**Sex, n (%)**			
	Female	51 (85)	
	Male	9 (15)	
Education: ≥12 y, n (%)			37 (62)
High blood pressure, n (%)			35 (58)
Obesity, n (%)			28 (47)
Dyslipidemia, n (%)			47 (78)
Tobacco use, n (%)			14 (23)
Hypothyroidism, n (%)			14 (23)
Mild vision problems, n (%)			60 (100)
Mild hearing problems, n (%)			1 (2)
Heart failure, n (%)			4 (7)
Chronic respiratory disease, n (%)			12 (20)
Diabetes, n (%)			14 (23)
Lives alone, n (%)			13 (22)
Has internet access at home, n (%)			57 (95)
Knows how to use a smartphone, n (%)			56 (93)
Has Android smartphone, n (%)			49 (82)

### Validation of the App’s Assessment

*To* validate the CVH categorization, we compared the NeoMayor app categorization and the face-to-face clinical assessment, which yielded a κ index of 0.51 (*P*<.001), indicating a moderate level of agreement between the 2 evaluations. In addition, the Pearson correlation between the 2 assessments (*r*=0.692; *P*=.001) indicated a good correlation for the total CVH index score. At baseline, our results indicate that 54 (90%) of the 60 participants had intermediate CVH, 4 (7%) had low CVH, and 4 (7%) had optimal CVH.

### Potential Effectiveness

**Figure 5:**
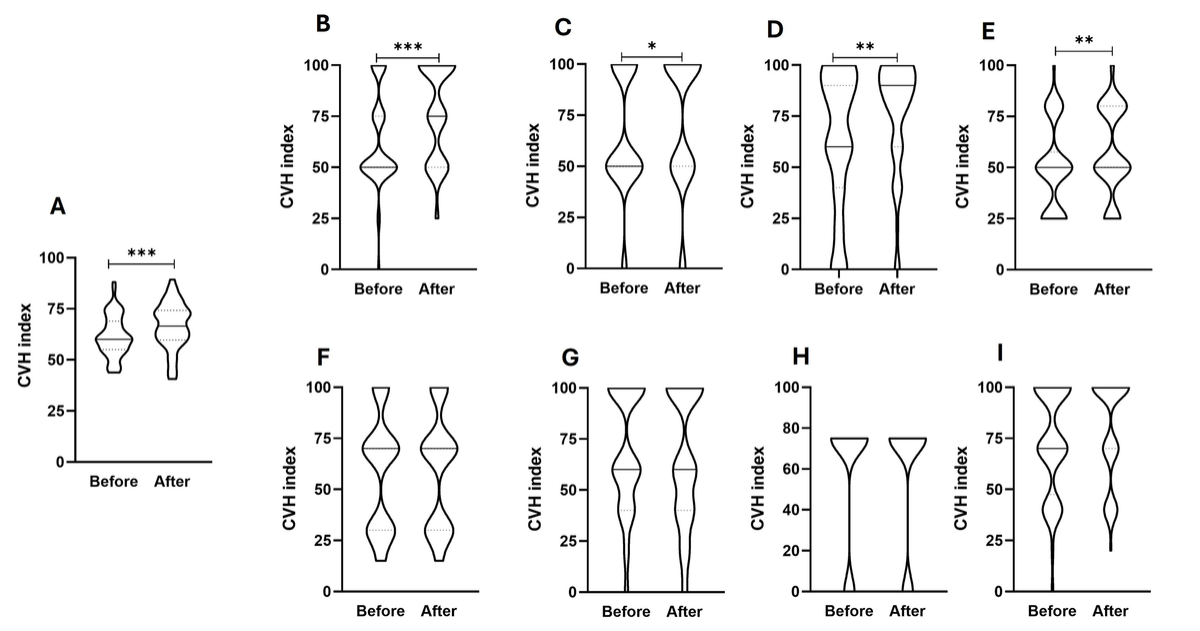
Changes in the cardiovascular health (CVH) index after NeoMayor use. (A) Total Life’s Essential 8. (B) Blood pressure. (C) Blood glucose level. (D) Physical activity. (E) Diet. (F) BMI. (G) Blood lipids. (H) Tobacco use. (I) Sleep. Violin plots compare the global CVH index and its subscores before and after NeoMayor use. The solid lines represent the median, and the dashed lines represent the quartiles. A paired sample t test compared CVH index differences. *P<.05, **P<.005, ***P<.001.

The average CVH index score at baseline was 64.3 (SD 10.8), which improved significantly to 69.0 (SD 11.03) after 2 months of NeoMayor app use (*P*<.001; Figure 5A), resulting in a moderate effect size (Cohen *d*=−0.548, −2.55 to 1.46). Participants showed significant improvements in 4 of the 8 subscores of the CVH index: blood pressure (Figure 5B), blood glucose level (Figure 5C), physical activity (Figure 5D), and diet (Figure 5E). However, no significant changes were observed in BMI, blood lipids, tobacco use, and sleep (Figures 5F-5I; Table 2). Indeed, the number of participants who smoked remained unchanged. Next, we classified the participants into those who showed improvement and those who did not: 32% (19/60) showed improvement. There were no significant predictors of improvement after testing the effects of age, sex, educational level, baseline depressive symptoms, and baseline CVH index, but there was a tendency for a negative effect of higher baseline depressive symptoms (odds ratio 0.69, 95% CI 0.48–1.00; *P*=.05) and healthier baseline CVH status (odds ratio 0.94, 95% CI 0.88–1.00; *P*=.06) on the possibility of improvement.

Regarding other results, we observed changes in anthropometric measurements, with an average weight reduction of 1 kg and a decrease in waist circumference of 7 cm, both indicative of improved body fat distribution. Physical performance showed improvement, with faster gait speed (an average reduction of 2 s), enhanced upper limb strength (measured by the handgrip test), increased lower limb strength (measured by the 5-repetition chair stand test), and improved SPPB scores. In addition, a significant reduction of 10 mm Hg in systolic blood pressure was noted. Blood tests revealed a decrease in high-density lipoprotein cholesterol levels, although low-density lipoprotein cholesterol and triglyceride levels showed nonsignificant increases. There were no differences in cognitive assessment or depressive symptoms, but there was an improvement in self-perceived memory after app use (Table 2).

**Table 2:** Comparison of variables assessed before and after the NeoMayor pilot study (n=60).

Variables		A: before pilot, mean (SD)	B: after pilot, mean (SD)	*P* value: A versus B^a^
**Physical examination**				
	Weight (kg)	72.0 (14.0)	71.0 (17.0)	.53
	Waist circumference (cm)	99.0 (11.0)	92.0 (12.0)	*<.001* ^b^
	Systolic blood pressure (mm Hg)	131.0 (17.0)	121.0 (14.0)	*<.001*
	Diastolic blood pressure (mm Hg)	72.0 (14.0)	72.0 (14.0)	.57
	Handgrip test (kg)	25.0 (6.0)	26.0 (6.0)	.48
	5-repetition chair stand test time (s)	10.0 (2.0)	8.0 (2.0)	*<.001*
	SPPB^c^ (score: 0-12)	11.3 (1.1)	11.9 (0.5)	*<.001*
	Timed up and go simple task (s)	10.0 (2.0)	8.0 (2.0)	*<.001*
	Timed up and go dual task (s)	11.0 (2.0)	9.0 (2.0)	*<.001*
**Blood tests**				
	Fasting blood glucose level	107.0 (26.0)	104.0 (24.0)	.85
	Fasting blood total cholesterol level	192.0 (36.0)	198.0 (43.0)	.17
	Fasting blood HDL^d^ cholesterol level	57.0 (15.0)	52.0 (14.0)	*.005*
	Fasting blood LDL^e^ cholesterol level	109.0 (34.0)	117.0 (39.0)	.45
	Fasting blood triglycerides level	134.0 (52.0)	146.0 (67.0)	.43
**Life’s Essential 8**				
	CVH^f^ index	63.0 (10.8)	69.0 (11.3)	*<.001*
	Blood pressure	63.7 (23.2)	77.5 (23.3)	*<.001*
	Blood lipids	69.0 (29.5)	65.0 (32.4)	.18
	Blood glucose	65.8 (29.8)	73.3 (32.5)	*.04*
	BMI	58.2 (28.2)	59.6 (28.1)	.38
	Physical activity	62.2 (34.4)	76.3 (27.4)	*.003*
	Diet	49.8 (21.1)	57.3 (21.6)	*.006*
	Tobacco use	58.7 (31.1)	58.7 (31.1)	>.99
	Sleep	73.8 (26.1)	79.6 (25.1)	.07
**Questionnaires and cognitive test scores**				
	MIND^g^ food score	8.0 (2.0)	8.0 (6.0)	.49
	IPAQ^h^ score	1.8 (0.6)	2.4 (0.6)	*<.001*
	PSQI^i^ (self-reported sleep duration)	6.4 (1.0)	6.4 (1.0)	.74
	PSQI score	8.0 (4.0)	8.0 (4.0)	.75
	MCA^j^	5.0 (3.0)	3.0 (2.0)	*.02*
	GDS^k^	2.0 (2.0)	2.0 (3.0)	.08
	MEFO^l^	12.0 (1.0)	12.0 (1.0)	.14

^a^Paired sample *t* test with equal variances.

^b^Italicization indicates values that met the significance threshold (*P*<.05).

^c^SPPB: Short Physical Performance Battery.

^d^HDL: high-density lipoprotein.

^e^LDL: low-density lipoprotein.

^f^CVH: cardiovascular health.

^g^MIND: Mediterranean-DASH Diet Intervention for Neurodegenerative Delay.

^h^IPAQ: International Physical Activity Questionnaire.

^i^PSQI: Pittsburgh Sleep Quality Index.

^j^MCA: self-perception of memory scales.

^k^GDS: Geriatric Depression Scale.

^l^MEFO: memory, fluency, and orientation test.

### Qualitative Results

#### Overview

The usability as well as barriers and facilitators to using the NeoMayor app were evaluated through an iterative process involving the clinical team and cocreation session participants. Among the participants, 88% (53/60) stated that their primary motivation for using the app was “to improve overall health,” followed by improving physical fitness and enhancing memory. In addition, 79% (47/60) of the participants considered improving their lifestyle especially important, and the same proportion believed that the app would help them achieve this goal. Regarding usability, 85% (51/60) of the participants found the app easy and intuitive to use. For the initial health survey, 90% (54/60) considered the personalized results delivered by the survey useful and easy to understand. In terms of user satisfaction, 66% (39/60) found the app highly engaging, and 64% (38/60) were very satisfied with their UX. Moreover, 63% (38/60) rated the app as highly motivating and found the app intuitive to use.

#### Adherence

To assess study adherence, we monitored app use during the first month. Participants spent an average of 12,242.6 (SD 2359.0) seconds using the app, equivalent to approximately 204.0 (SD 5.9) minutes. This corresponds to approximately 400 seconds (6.7 min) of app use per day.

## Discussion

### Principal Findings

In this study, we described the design, development, and validation of NeoMayor, the first mHealth app aimed at promoting healthy lifestyles as well as cardiovascular and brain health in older adults in Chile through multidomain healthy lifestyle interventions. The NeoMayor app categorizes users’ CVH profiles based on a survey inspired by Life’s Essential 8, which was adapted and validated in this pilot study. The app then delivers personalized recommendations and content covering physical activity, nutrition, mental health, memory, and general health management for healthy brain promotion [66], providing a personalized guide to healthier living. This structured and systematic development process, combined with the validation of the content and design, allows for potential replication in other mHealth apps, particularly for populations with limited digital literacy or access to health technologies [52]. These findings also align with previously described barriers and facilitators to adopting mHealth-mediated strategies [21,26,27].

Although several international initiatives exist to promote health care in older adults, most have been developed in high-income countries and are not designed for populations with lower educational levels, who require special adaptations and cultural validation to meet their needs [67]. In this context, NeoMayor is the first app specifically adapted for Chilean older adults to support integral health care promotion.

Regarding adherence, the average daily use time of the NeoMayor app was notably higher (6.7 min/d) than that reported in other studies of older adults using mobile apps (2 min/d), indicating a high level of engagement [68], which could be associated with the participants’ high technological skills and motivation. One important factor to consider in the adoption of mHealth technology is technology-related anxiety [69] in people with limited digital literacy, which may require positive management to encourage app use [17]. Although this study included participants with functional independence, cognitive ability, and basic digital skills, broader implementation for older people with low digital literacy or cognitive issues [70] may require alternative strategies. These could include health care professional– or coach-assisted mHealth interventions to overcome moderate mHealth literacy or social digital barriers to engagement and empower health literacy [71].

The findings of the pilot study suggest that using the NeoMayor app led to improvements in both metabolic health and lifestyle habits, which are key indicators of the app’s potential effectiveness [72]. The improvements observed in blood pressure (a reduction of 10 mm Hg in systolic blood pressure), blood glucose levels, physical activity, and diet—key elements in the prevention of cardiovascular disease and other NCDs—represent a relevant finding that should be confirmed in a larger-scale RCT [65]. Although we did not find significant changes in BMI, participants experienced a significant reduction in waist circumference, which is more closely associated with cardiovascular risk than BMI [73]. This is a very important result, considering that 47% (28/60) of the participants were obese.

The significant enhancement in functional physical performance, measured by the SPPB [74], gait speed alone, and the 5-repetition chair stand test alone, is relevant, considering its critical importance in reducing the risk of physical fragility and falls in older adults [75]. Moreover, the 2-second reduction in the 5-repetition chair stand test represents a statistically significant and clinically relevant change among older Chileans, which is associated with higher survival rates [76]. These improvements in physical performance may be attributed to the physical activity module, which includes instructional videos, consistent with findings from previous research on the impact of video-guided physical activity interventions [52,77]. In addition, these improvements contribute to enhanced metabolic health. Furthermore, we found an increase in self-perceived memory, which could be associated with both physical activity and the global cognitive activity promoted by the use of the app. Previous studies report that physical activity combined with cognitive training can improve self-perceived memory in older adults [78], and exercise is a main component of cognitive health [79].

As an additional strength, usability testing demonstrated easy user navigation, with a main menu that provided intuitive access to the app’s modules. Participants reported a preference for challenge-based navigation, which allowed them to set specific health goals and stay motivated. However, minor modifications should be addressed in future iterations of the app, including more engaging navigation elements, considering the importance of structural measures to support lifestyle changes [14,80].

### Limitations

This study included only participants without sensory or cognitive impairments and with good technological skills. Future adaptations could expand the intervention’s applicability by considering the co-design of an inclusive version of the app tailored to varying levels of digital literacy. In addition, because most of the participants were female—a common proportion in similar studies [81]—the generalizability validity of our findings may be limited. This is a frequent issue in pilot studies, where feasibility is the main objective, and could be overcome in future studies using the NeoMayor app in population-based samples. As a pilot study, the methodology used cannot establish the efficacy of NeoMayor in CVH independent of confounding factors, but it provides a solid foundation for a subsequent RCT with strategies to overcome sample bias. Indeed, with the implementation of the design improvements, the NeoMayor app has reached technology readiness level 4 (Figure 2). The next phase will involve evaluating the effectiveness of NeoMayor on CVH and lifestyle in an RCT, which should include at least 200 participants with different digital literacy and technological skills, based on sample size estimation considering the moderate effect size with regard to CVH observed in this pilot.

### Conclusions

This study described the design, development, and validation of a comprehensive mHealth app that aims to promote healthy lifestyles as well as cardiovascular and brain health in older adults in Chile. Using a person-centered, multidisciplinary approach grounded in lean UX and agile methodology for co-design and codevelopment, NeoMayor demonstrated good feasibility and potential effectiveness in improving CVH and physical performance, which will be evaluated soon in a larger RCT. These findings indicate that the NeoMayor app can provide an accessible, affordable, and implementable strategy to support healthy aging.

## Abbreviations

CVH: cardiovascular health
ICOPE: Integrated Care for Older People
mHealth: mobile health
NCD: noncommunicable disease
RCT: randomized controlled trial
RE-AIM: Reach, Effectiveness, Adoption, Implementation, and Maintenance
REDCap: Research Electronic Data Capture
SPPB: Short Physical Performance Battery
UX: user experience
WHO: World Health Organization

## References

[1] Moreno X, Lera L, Albala C (2020). Disability-free life expectancy and life expectancy in good self-rated health in Chile: gender differences and compression of morbidity between 2009 and 2016. PLoS One.

[2] Albala C, Sánchez H, Lera L, Angel B, Cea X (2011). Socioeconomic inequalities in active life expectancy and disability related to obesity among older people. Rev Med Chil.

[3] (2024). Global burden of disease 2021: findings from the GBD 2021 Study. Institute for Health Metrics and Evaluation.

[4] Sousa RM, Ferri CP, Acosta D, Albanese E, Guerra M, Huang Y, Jacob KS, Jotheeswaran A, Rodriguez JJ, Pichardo GR, Rodriguez MC, Salas A, Sosa AL, Williams J, Zuniga T, Prince M (2009). Contribution of chronic diseases to disability in elderly people in countries with low and middle incomes: a 10/66 Dementia Research Group population-based survey. The Lancet.

[5] Paradela RS, Calandri I, Castro NP, Garat E, Delgado C, Crivelli L, Yaffe K, Ferri CP, Mukadam N, Livingston G, Suemoto CK (2024). Population attributable fractions for risk factors for dementia in seven Latin American countries: an analysis using cross-sectional survey data. The Lancet Glob Health.

[6] Roth GA, Mensah GA, Fuster V (2020). The global burden of cardiovascular diseases and risks: a compass for global action. J Am Coll Cardiol.

[7] Baker LD, Snyder HM, Espeland MA, Whitmer RA, Kivipelto M, Woolard N, Katula J, Papp KV, Ventrelle J, Graef S, Hill MA, Rushing S, Spell J, Lovato L, Felton D, Williams BJ, Ghadimi Nouran M, Raman R, Ngandu T, Solomon A, Wilmoth S, Cleveland ML, Williamson JD, Lambert KL, Tomaszewski Farias S, Day CE, Tangney CC, Gitelman DR, Matongo O, Reynolds T, Pavlik VN, Yu MM, Alexander AS, Elbein R, McDonald AM, Salloway S, Wing RR, Antkowiak S, Morris MC, Carrillo MC (2024). Study design and methods: U.S. study to protect brain health through lifestyle intervention to reduce risk (U.S. POINTER). Alzheimers Dement.

[8] Mukadam N, Wolters FJ, Walsh S, Wallace L, Brayne C, Matthews FE, Sacuiu S, Skoog I, Seshadri S, Beiser A, Ghosh S, Livingston G (2024). Changes in prevalence and incidence of dementia and risk factors for dementia: an analysis from cohort studies. The Lancet Public Health.

[9] Ngandu T, Lehtisalo J, Solomon A, Levälahti E, Ahtiluoto S, Antikainen R, Bäckman L, Hänninen T, Jula A, Laatikainen T, Lindström J, Mangialasche F, Paajanen T, Pajala S, Peltonen M, Rauramaa R, Stigsdotter-Neely A, Strandberg T, Tuomilehto J, Soininen H, Kivipelto M (2015). A 2 year multidomain intervention of diet, exercise, cognitive training, and vascular risk monitoring versus control to prevent cognitive decline in at-risk elderly people (FINGER): a randomised controlled trial. The Lancet.

[10] Kivipelto M (2019). World-wide fingers network: a global approach to risk reduction and prevention of dementia. J Neurol Sci.

[11] Crivelli L, Calandri IL, Suemoto CK, Salinas RM, Velilla LM, Yassuda MS, Caramelli P, Lopera F, Nitrini R, Sevlever GE, Sosa AL, Acosta D, Baietti AM, Cusicanqui MI, Custodio N, De Simone SD, Derio CD, Duque-Peñailillo L, Duran JC, Jiménez-Velázquez IZ, Leon-Salas JM, Bergamo Y, Clarens MF, Damian A, Demey I, Helou MB, Márquez C, Martin ME, Martin MD, Querze D, Surace EI, Acosta-Egea S, Aguirre-Salvador E, de Souza LC, Cançado GH, Brucki SM, Friedlaender CV, Gomes KB, Gutierrez M, Ríos CL, Galindo JG, Montesinos R, Nuñez-Herrera A, Ospina-Henao S, Rodríguez G, Masson VR, Sánchez M, Schenk CE, Soto L, Barbosa MT, Tosatti JA, Vicuña Y, Espeland M, Hakansson K, Kivipelto M, Baker L, Snyder H, Carrillo M, Allegri RF (2023). Latin American Initiative for Lifestyle Intervention to Prevent Cognitive Decline (LatAm-FINGERS): study design and harmonization. Alzheimers Dement.

[12] Parra-Soto S, Leiva-Ordoñez AM, Troncoso-Pantoja C, Matus-Castillo C, Petermann-Rocha F, Martínez-Sanguinetti MA, Martorell M, Ulloa N, Concha-Cisternas Y, Cigarroa I, Villagrán M, Mardones L, Laserre-Laso N, Celis-Morales C (2021). Association of adiposity and diabetes mellitus type 2 by education level in the Chilean population. Rev Med Chil.

[13] (2018). Encuesta Nacional de Salud 2016-2017: Primeros resultados. Ministerio de Salud (Minsal), Subsecretaría de Salud Pública, División de Planificación Sanitaria, Departamento de Epidemiología.

[14] Valentino G, Vio F, Rodriguez-Osiac L (2023). Analysis of the Chilean health promotion policy "choose a healthy lifestyle". Rev Med Chil.

[15] Legaz A, Altschuler F, Gonzalez-Gomez R, Hernández H, Baez S, Migeot J, Fittipaldi S, Medel V, Maito MA, Godoy ME, Moguilner S, Cruzat J, Coronel-Oliveros C, Tagliazuchi E, Santamaria Garcia H, Farina FR, Reyes P, Javandel S, García AM, Deleglise Á, Matallana DL, Avila-Funes JA, Slachevsky A, Behrens MI, Custodio N, Trujillo-Llano C, Cardona JF, Barttfeld P, Brusco IL, Bruno MA, Sosa Ortiz AL, Pina-Escudero SD, Takada LT, França Resende EP, Possin KL, Okada de Oliveira M, Hu K, Lopera F, Lawlor B, Valcour V, Yokoyama JS, Miller B, Ibañez A (2025). Structural inequality linked to brain volume and network dynamics in aging and dementia across the Americas. Nat Aging.

[16] Richard E, Moll van Charante EP, Hoevenaar-Blom MP, Coley N, Barbera M, van der Groep A, Meiller Y, Mangialasche F, Beishuizen CB, Jongstra S, van Middelaar T, Van Wanrooij LL, Ngandu T, Guillemont J, Andrieu S, Brayne C, Kivipelto M, Soininen H, Van Gool WA (2019). Healthy ageing through internet counselling in the elderly (HATICE): a multinational, randomised controlled trial. The Lancet Digital Health.

[17] Hafdi M, Eggink E, Hoevenaar-Blom MP, Witvliet MP, Andrieu S, Barnes L, Brayne C, Brooks R, Coley N, Georges J, van der Groep A, van Marwijk H, van der Meijden M, Song L, Song M, Wang Y, Wang W, Wang W, Wimo A, Ye X, Moll van Charante EP, Richard E (2021). Design and development of a mobile health (mHealth) platform for dementia prevention in the prevention of dementia by mobile phone applications (PRODEMOS) project. Front Neurol.

[18] mHealth: new horizons for health through mobile technologies. World Health Organization.

[19] Van Charante EM, Hoevenaar-Blom MP, Song M, Andrieu S, Coley N, Georges J, Van Gool WA, Handels R, Wang Y, Wimo A, Barnes CB, Wang W, Richard E (2024). Prevention of dementia using mobile phone applications (PRODEMOS): a multinational, randomised, controlled effectiveness-implementation trial. Lancet Healthy Longev.

[20] Kim S, Gajos KZ, Muller M, Grosz BJ (2016). Acceptance of mobile technology by older adults: a preliminary study. Proceedings of the 18th International Conference on Human-Computer Interaction with Mobile Devices and Services.

[21] Wilson J, Heinsch M, Betts D, Booth D, Kay-Lambkin F (2021). Barriers and facilitators to the use of e-health by older adults: a scoping review. BMC Public Health.

[22] Eggink E, Hafdi M, Hoevenaar-Blom MP, Song M, Andrieu S, Barnes LE, Birck C, Brooks RL, Coley N, Ford E, Georges J, van der Groep A, van Gool WA, Handels R, Hou H, Li D, Liu H, Lyu J, van Marwijk H, van der Meijden M, Niu Y, Sadhwani S, Wang W, Wang Y, Wimo A, Ye X, Yu Y, Zeng Q, Zhang W, Wang W, Brayne C, Moll van Charante EP, Richard E (2021). Prevention of dementia using mobile phone applications (PRODEMOS): protocol for an international randomised controlled trial. BMJ Open.

[23] Barreda Gutiérrez M, Cantarero-Prieto D, Pascual Sáez M (2024). Age, technology, and the digital divide: are they directly related to mental health problems?. Healthcare (Basel).

[24] Cajita MI, Hodgson NA, Lam KW, Yoo S, Han HR (2018). Facilitators of and barriers to mHealth adoption in older adults with heart failure. Comput Inform Nurs.

[25] Mitzner TL, Boron JB, Fausset CB, Adams AE, Charness N, Czaja SJ, Dijkstra K, Fisk AD, Rogers WA, Sharit J (2010). Older adults talk technology: technology usage and attitudes. Comput Human Behav.

[26] Wildenbos GA, Peute L, Jaspers M (2018). Aging barriers influencing mobile health usability for older adults: a literature based framework (MOLD-US). Int J Med Inform.

[27] Mace RA, Mattos MK, Vranceanu AM (2022). Older adults can use technology: why healthcare professionals must overcome ageism in digital health. Transl Behav Med.

[28] Jongstra S, Beishuizen C, Andrieu S, Barbera M, van Dorp M, van de Groep B, Guillemont J, Mangialasche F, van Middelaar T, Moll van Charante E, Soininen H, Kivipelto M, Richard E (2017). Development and validation of an interactive internet platform for older people: the healthy ageing through internet counselling in the elderly study. Telemed J E Health.

[29] Observatorio del Envejecimiento (2022). Uso de internet y tecnologías de la información y comunicación en las personas mayores. Centro de Estudios de Vejez y Envejecimiento de la Pontificia Universidad Católica de Chile y Compañía de Seguros Confuturo.

[30] (2025). Chile y Sus Mayores: Sexta Encuesta Nacional de Calidad de Vida en la Vejez 2022 UC-Caja Los Andes. Pontificia Universidad Católica de Chile.

[31] Or CK, Holden RJ, Valdez RS, Duffy VG, Ziefle M, Rau PL, Tseng MM (2022). Human factors engineering and user-centered design for mobile health technology: enhancing effectiveness, efficiency, and satisfaction. Human-Automation Interaction.

[32] Alruwaili MM, Shaban M, Elsayed Ramadan OM (2023). Digital health interventions for promoting healthy aging: a systematic review of adoption patterns, efficacy, and user experience. Sustainability.

[33] Borges do Nascimento IJ, Abdulazeem H, Vasanthan LT, Martinez EZ, Zucoloto ML, Østengaard L, Azzopardi-Muscat N, Zapata T, Novillo-Ortiz D (2023). Barriers and facilitators to utilizing digital health technologies by healthcare professionals. NPJ Digit Med.

[34] Gutiérrez M (2025). BioRender: Scientific image and illustration software.

[35] LaMonica HM, Roberts AE, Lee GY, Davenport TA, Hickie IB (2021). Privacy practices of health information technologies: privacy policy risk assessment study and proposed guidelines. J Med Internet Res.

[36] (2020). Integrated care for older people (‎‎‎‎‎‎‎‎‎ICOPE)‎‎‎‎‎‎‎‎‎: guidance for person-centered assessment and pathways in primary care. Pan American Health Organization.

[37] (2019). Risk reduction of cognitive decline and dementia: WHO guidelines. World Health Organization.

[38] Primary or essential arterial hypertension in people 15 years of age and older. Superintendencia de Salud.

[39] Lloyd-Jones DM, Allen NB, Anderson CA, Black T, Brewer LC, Foraker RE, Grandner MA, Lavretsky H, Perak AM, Sharma G, Rosamond W (2022). Life's essential 8: updating and enhancing the American Heart Association's construct of cardiovascular health: a presidential advisory from the American Heart Association. Circulation.

[40] Howard G, Cushman M, Blair J, Wilson NR, Yuan Y, Safford MM, Levitan EB, Judd SE, Howard VJ (2024). Comparative discrimination of life's simple 7 and life's essential 8 to stratify cardiovascular risk: is the added complexity worth it?. Circulation.

[41] Clocchiatti-Tuozzo S, Rivier CA, Renedo D, Huo S, Hawkes MA, de Havenon A, Schwamm LH, Sheth KN, Gill TM, Falcone GJ (2024). Life's essential 8 and poor brain health outcomes in middle-aged adults. Neurology.

[42] Abad Andrades MS, Gálvez Pérez MJ, Jana Hoebel D, Vergara Andueza O (2021). Guía práctica salud mental y bienestar para personas mayores. SENAMA, Ministerio de Desarrollo Social y Familia, Gobierno de Chile.

[43] (2024). Plan nacional de Salud mental 2017-2025. Biblioteca del Congreso Nacional de Chile.

[44] (2020). WHO guidelines on physical activity and sedentary behaviour. World Health Organization.

[45] (2024). Fiscal policies to promote healthy diets: WHO guideline. World Health Organization.

[46] Mess EV, Kramer F, Krumme J, Kanelakis N, Teynor A (2024). Use of creative frameworks in health care to solve data and information problems: scoping review. JMIR Hum Factors.

[47] Hodson N, Woods P, Sobolev M, Giacco D (2024). A digital microintervention supporting evidence-based parenting skills: development study using the agile scrum methodology. JMIR Form Res.

[48] Riede M, Breitschwerdt R, Liebe JD (2024). Inclusive design thinking for the development of digital health applications: a methodology review. Stud Health Technol Inform.

[49] Vercruyssen A, Schirmer W, Geerts N, Mortelmans D (2023). How “basic” is basic digital literacy for older adults? Insights from digital skills instructors. Front Educ.

[50] Gothelf J, Seiden J (2013). Lean UX: Applying Lean Principles to Improve User Experience.

[51] Gonen E (2019). Tim Brown, change by design: how design thinking transforms organizations and inspires innovation (2009). Mark Global Dev Rev.

[52] Daniels K, Lemmens R, Knippenberg E, Marinus N, Vonck S, Baerts J, Bergs J, Spooren A, Hansen D, Bonnechère B (2023). Promoting physical activity and a healthy active lifestyle in community-dwelling older adults: a design thinking approach for the development of a mobile health application. Front Public Health.

[53] Gutiérrez M (2025). BioRender: Scientific image and illustration software.

[54] Or C, Tao D (2012). Usability study of a computer-based self-management system for older adults with chronic diseases. JMIR Res Protoc.

[55] Gutiérrez M (2025). BioRender: Scientific image and illustration software.

[56] Delgado Derio C, Guerrero Bonnet S, Troncoso Ponce M, Araneda Yañez A, Slachevsky Chonchol A, Behrens Pellegrino MI (2013). Memory, fluency, and orientation (MEFO): a five-minute screening test for cognitive decline. Neurología (English Edition).

[57] Buysse DJ, Reynolds CF 3rd, Monk TH, Berman SR, Kupfer DJ (1989). The Pittsburgh Sleep Quality Index: a new instrument for psychiatric practice and research. Psychiatry Res.

[58] Morris MC, Tangney CC, Wang Y, Sacks FM, Barnes LL, Bennett DA, Aggarwal NT (2015). MIND diet slows cognitive decline with aging. Alzheimers Dement.

[59] Craig CL, Marshall AL, Sjöström M, Bauman AE, Booth ML, Ainsworth BE, Pratt M, Ekelund U, Yngve A, Sallis JF, Oja P (2003). International physical activity questionnaire: 12-country reliability and validity. Med Sci Sports Exerc.

[60] Folstein MF, Folstein SE, McHugh PR (1975). "Mini-mental state". A practical method for grading the cognitive state of patients for the clinician. J Psychiatr Res.

[61] Yesavage JA, Brink TL, Rose TL, Lum O, Huang V, Adey M, Leirer VO (1982). Development and validation of a geriatric depression screening scale: a preliminary report. J Psychiatr Res.

[62] Zelinski EM, Gilewski MJ (2004). A 10-item Rasch modeled memory self-efficacy scale. Aging Ment Health.

[63] Guralnik JM, Simonsick EM, Ferrucci L, Glynn RJ, Berkman LF, Blazer DG, Scherr PA, Wallace RB (1994). A short physical performance battery assessing lower extremity function: association with self-reported disability and prediction of mortality and nursing home admission. J Gerontol.

[64] Ory MG, Altpeter M, Belza B, Helduser J, Zhang C, Smith ML (2014). Perceived utility of the RE-AIM framework for health promotion/disease prevention initiatives for older adults: a case study from the U.S. evidence-based disease prevention initiative. Front Public Health.

[65] Thabane L, Ma J, Chu R, Cheng J, Ismaila A, Rios LP, Robson R, Thabane M, Giangregorio L, Goldsmith CH (2010). A tutorial on pilot studies: the what, why and how. BMC Med Res Methodol.

[66] Rost NS, Salinas J, Jordan JT, Banwell B, Correa DJ, Said RR, Selwa LM, Song S, Evans DA (2023). The brain health imperative in the 21st century—a call to action. Neurology.

[67] Liu N, Yin J, Tan SS, Ngiam KY, Teo HH (2021). Mobile health applications for older adults: a systematic review of interface and persuasive feature design. J Am Med Inform Assoc.

[68] Sweeney M, Barton W, Nebeker C (2023). Evaluating mobile apps targeting older adults: descriptive study. JMIR Form Res.

[69] Hoque R, Sorwar G (2017). Understanding factors influencing the adoption of mHealth by the elderly: an extension of the UTAUT model. Int J Med Inform.

[70] Gutiérrez M, Mauro J, Asecio J, Acevedo F, Herrada J, Torres C, Delgado C, Fasce G (2023). Implementation of a virtual telehealth course for Chilean older adults and health students. Rev Med Chil.

[71] Kim M, Kim B, Park S (2024). Social support, eHealth literacy, and mhealth use in older adults with diabetes: moderated mediating effect of the perceived importance of app design. Comput Inform Nurs.

[72] Lehtisalo J, Rusanen M, Solomon A, Antikainen R, Laatikainen T, Peltonen M, Strandberg T, Tuomilehto J, Soininen H, Kivipelto M, Ngandu T (2022). Effect of a multi-domain lifestyle intervention on cardiovascular risk in older people: the FINGER trial. Eur Heart J.

[73] Ross R, Neeland IJ, Yamashita S, Shai I, Seidell J, Magni P, Santos RD, Arsenault B, Cuevas A, Hu FB, Griffin BA, Zambon A, Barter P, Fruchart J, Eckel RH, Matsuzawa Y, Després JP (2020). Waist circumference as a vital sign in clinical practice: a Consensus Statement from the IAS and ICCR Working Group on Visceral Obesity. Nat Rev Endocrinol.

[74] Guralnik JM, Ferrucci L, Pieper CF, Leveille SG, Markides KS, Ostir GV, Studenski S, Berkman LF, Wallace RB (2000). Lower extremity function and subsequent disability: consistency across studies, predictive models, and value of gait speed alone compared with the short physical performance battery. J Gerontol A Biol Sci Med Sci.

[75] Dent E, Morley JE, Cruz-Jentoft AJ, Woodhouse L, Rodríguez-Mañas L, Fried LP, Woo J, Aprahamian I, Sanford A, Lundy J, Landi F, Beilby J, Martin FC, Bauer JM, Ferrucci L, Merchant RA, Dong B, Arai H, Hoogendijk EO, Won CW, Abbatecola A, Cederholm T, Strandberg T, Gutiérrez Robledo LM, Flicker L, Bhasin S, Aubertin-Leheudre M, Bischoff-Ferrari HA, Guralnik JM, Muscedere J, Pahor M, Ruiz J, Negm AM, Reginster JY, Waters DL, Vellas B (2019). Physical frailty: ICFSR international clinical practice guidelines for identification and management. J Nutr Health Aging.

[76] Albala C, Lera L, Sanchez H, Angel B, Márquez C, Arroyo P, Fuentes P (2017). Frequency of frailty and its association with cognitive status and survival in older Chileans. Clin Interv Aging.

[77] Blair CK, Harding E, Wiggins C, Kang H, Schwartz M, Tarnower A, Du R, Kinney AY (2021). A home-based mobile health intervention to replace sedentary time with light physical activity in older cancer survivors: randomized controlled pilot trial. JMIR Cancer.

[78] Miller KJ, Siddarth P, Gaines JM, Parrish JM, Ercoli LM, Marx K, Ronch J, Pilgram B, Burke K, Barczak N, Babcock B, Small GW (2012). The memory fitness program: cognitive effects of a healthy aging intervention. Am J Geriatr Psychiatry.

[79] Suzuki T, Shimada H, Makizako H, Doi T, Yoshida D, Ito K, Shimokata H, Washimi Y, Endo H, Kato T (2013). A randomized controlled trial of multicomponent exercise in older adults with mild cognitive impairment. PLoS One.

[80] Recio-Rodríguez JI, Lugones-Sanchez C, Agudo-Conde C, González-Sánchez J, Tamayo-Morales O, Gonzalez-Sanchez S, Fernandez-Alonso C, Maderuelo-Fernandez JA, Mora-Simon S, Gómez-Marcos MA, Rodriguez-Sanchez E, Garcia-Ortiz L (2019). Combined use of smartphone and smartband technology in the improvement of lifestyles in the adult population over 65 years: study protocol for a randomized clinical trial (EVIDENT-Age study). BMC Geriatr.

[81] Lyons EJ, Swartz MC, Lewis ZH, Martinez E, Jennings K (2017). Feasibility and acceptability of a wearable technology physical activity intervention with telephone counseling for mid-aged and older adults: a randomized controlled pilot trial. JMIR Mhealth Uhealth.

